# Analysis of *in vitro *bioactivity data extracted from drug discovery literature and patents: Ranking 1654 human protein targets by assayed compounds and molecular scaffolds

**DOI:** 10.1186/1758-2946-3-14

**Published:** 2011-05-13

**Authors:** Christopher Southan, Kiran Boppana, Sarma ARP Jagarlapudi, Sorel Muresan

**Affiliations:** 1 DECS Global Compound Sciences, Computational Chemistry, AstraZeneca R&D Mölndal, S-431 83 Mölndal, Sweden; 2 GVK Biosciences Pvt. Ltd., S1, Phase-1, Technocrats Industrial Estate, Balanagar, Hyderabad, 500 037, India

## Abstract

**Background:**

Since the classic Hopkins and Groom druggable genome review in 2002, there have been a number of publications updating both the hypothetical and successful human drug target statistics. However, listings of research targets that define the area between these two extremes are sparse because of the challenges of collating published information at the necessary scale. We have addressed this by interrogating databases, populated by expert curation, of bioactivity data extracted from patents and journal papers over the last 30 years.

**Results:**

From a subset of just over 27,000 documents we have extracted a set of compound-to-target relationships for biochemical *in vitro *binding-type assay data for 1,736 human proteins and 1,654 gene identifiers. These are linked to 1,671,951 compound records derived from 823,179 unique chemical structures. The distribution showed a compounds-per-target average of 964 with a maximum of 42,869 (Factor Xa). The list includes non-targets, failed targets and cross-screening targets. The top-278 most actively pursued targets cover 90% of the compounds. We further investigated target ranking by determining the number of molecular frameworks and scaffolds. These were compared to the compound counts as alternative measures of chemical diversity on a per-target basis.

**Conclusions:**

The compounds-per-protein listing generated in this work (provided as a supplementary file) represents the major proportion of the human drug target landscape defined by published data. We supplemented the simple ranking by the number of compounds assayed with additional rankings by molecular topology. These showed significant differences and provide complementary assessments of chemical tractability.

## Introduction

An important factor in assessing the global progress in drug research is the number of targets for which therapeutic small-molecule modulators have been, are being, or could be, generated. This question was addressed in the landmark publication in 2002 that introduced the "druggable genome" concept [1].

This total of approximately 3,000 human proteins was arrived at by homologous family extrapolation from the targets of approved drugs at that time. The count of successful targets was updated in 2006 and stood then at 324, of which the subset of human proteins was 207 [2]. Despite many publications covering this topic, the inclusion of explicit listings of target identifiers, extrinsic to the data sets from which they were derived, are rare, with the partial exception of a poster that included 185 human targets of approved oral drugs [2].

Notwithstanding, there are now public databases from which it is possible to browse and extract targets with explicit links to bioactive compounds. DrugBank is one such resource [3]. It has a total of 6,827 drug entries including 1,431 FDA-approved small molecule drugs and 5,212 research compounds linked to 4,477 non-redundant protein sequences. These include primary targets, cross-screening targets, metabolising enzymes and associations inferred from compound name with protein name co-occurrences automatically extracted from the literature. The Therapeutic Targets Database (TTD) contains conceptually similar information to DrugBank but organised into a different data structure [4]. It provides sequence subsets of their total of 1,675 targets divided into 348 approved, 260 clinical trial and 1,067 research targets. The BindingDB resource also includes approved and research targets with a focus on measured small-molecule binding affinities and ligands. It currently includes 5,526 protein targets and 271,419 compounds [5]. The largest public resource of this type is the ChEMBL database with 8,091 targets and 658,075 compounds extracted from medicinal chemistry journal papers (N.B. a subset of ChEMBL data is now incorporated into BindingDB) [6]. Three of the databases above, DrugBank, TTD and ChEMBL, have recently been included in a comparative study of compounds and targets [7].

## Databases and Processing

The company GVKBIO [8] has developed a suite of databases over the last 9 years that are now unified under a single query interface, termed GVKBIO Online Structure Activity Relationships (GOSTAR) [9,10]. The results we present are from two of the six GOSTAR components, the Medicinal Chemistry (MCD) and Target (TGD) Databases. Their combined utility for mining drug research data has already been described [11-14]. In addition, the comparison of compound and target content of these with other bioactivity databases has been reported in publications that included the expansion of coverage between 2006 and 2008 [15,16].

**Figure 1:**
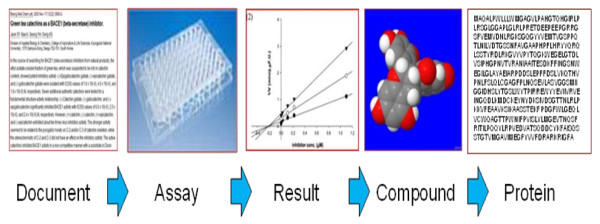
**Depiction of the key entities and the relationships between them (D-A-R-C-P) used to populate the MCD and TGD databases**.

The data in MCD and TGD are derived from the large-scale expert extraction of structure-activity relationships (SAR) from patents and journal papers reporting the results of drug discovery research [9]. The basic process is familiar to scientists working in this area. By inspecting a document "D" they can identify the description of a biochemical assay "A" (e.g. for enzyme activity) with a quantitative result "R" (e.g. a Ki) for a compound "C" (e.g. a specific chemical structure) that defines it as an activity modulator (e.g. an inhibitor) of protein target "P" (e.g. a protease). An outline of these relationships is shown in Figure 1.

At GVKBIO the relationships between these five entities of document, assay description, assay result, compound structure and protein target (D-A-R-C-P) are manually abstracted by a team of expert curators and transferred to document-centric relational databases. These contain data predominantly from the research phases of drug discovery but, because this extends back over 30 years, much of the primary data for approved drugs is included. The difference between them is that MCD extracts data from 120 journals selected for their high content of D-A-R-C-P relationships on a per-journal basis. TGD extracts the same relationships from patents covering the "big ten" target classes (kinases, GPCRs, proteases, nuclear hormone receptors, ion-channels, transporters, lipases, phosphatases, oxidoreductases and transferases). The process involves a triage to select a representative of the patent family for extraction. The addition of compounds to the database is limited to exemplified structures linked to quantitative or qualitative assay data. While all structures with quantitative results are extracted, where the activity data is ranged or only qualitative, the number of compounds extracted is capped at 200 or 100 examples, respectively [10].

Details of these databases are described elsewhere but briefly, structures and related metadata for the GOSTAR database records are stored in an Oracle database [17]. The compound counts are defined by a unique structure identifier based on the Standard InChIKey [18]. Protein information was added using NCBI Entrez Gene as primary source for protein (gene) names and identifiers (EGID) [19]. Where documents specified distinct alternative splice forms in assays, the common name used by the authors for that splice form was included with the EGID.

Target classes were assigned according to an internal schema. GVKBIO internally developed tools were also used to generate frameworks, scaffolds, and graph skeletons. The data was mined by running SQL queries against MCD and TGD subsets of the GOSTAR database. Additional filters were species, targets having an Entrez Gene name and assay type. Tables and graphs were generated in Excel.

## Results and Discussion

The content statistics of the aggregated MCD and TGD sources, with combined and separate numbers for patents and journal papers, are shown in Table 1.

**Table 1:** Content statistics and stringency triages for the combination of MCD and TGD.

Entity Type	Count
Total records	4442492
Unique compound structures	2856336
Unique compound structures from patents	2118101
Unique compound structures from journals	846026
Total quantitative assay results	10294189
Quantitative assay results from papers	5149097
Quantitative assay results from patents	5145092
Total documents	127330
Journal articles	79487
Patents	47843
Type-B assay results	4841851
Target names (all species) with type-B assay results	5334
Protein identifiers (all species) with type-B assay results	4043
Human proteins with type-B assay results	1736
Human gene identifiers with type-B assay results	1654
Unique compounds linked to human protein identifiers with type-B assay results	823179

The following aspects can be expanded. The average redundancy (records-per-unique structure) is 1.5 because some compounds, particularly reference reagents and established drugs, have assay data included in many documents. The predominant assay type is termed "type-B" or binding assay because it encompasses the enzyme inhibition and receptor binding assays most commonly reported for compounds tested against molecular targets *in vitro *and, implicitly, with binding specificity. The last three rows show the stringency used to define the final target listing. The target names in row 12 encompass both defined and undefined molecular targets (e.g. protein complexes or unresolved subfamilies) that are linked to compounds via a type-B assay result. These are further restricted in row 13 to only those molecular targets mapping to a protein identifier (e.g. an Entrez Gene ID or a Swiss-Prot accession). We added a final restriction to human sequences (row 14). We made this simplification choice for two reasons. The first was to exclude the many proteins used as cross-screening targets from mouse, rat and other model organisms. The second reason is that resolving anti-infective molecular target protein IDs also comes up against the problem of orthologous redundancy due to the multiplicity of viral, as well as bacterial sub-types, strains and species.

### Counting Distinct Human Protein Targets

We used the MCD and TGD databases to compile a list of all human gene identifiers that were linked with compounds via the results of type-B assays. The full list of these protein sequence identifiers, compound counts and document counts is included in Additional file 1, with the target statistics in Table 2.

**Table 2:** Protein Identifier Content for Additional file 1

Entity type	Count
Distinct protein names	1736
Entrez Gene ID (EGIDs)	1654
Symbols	1654
Symbol matching HGNC	1638
Splice form names	135
EGIDs with Splice forms	48

To maximise the curatorial specificity of mapping compounds to protein sequences, a number of splice form designations are includes where these names have been used in assays descriptions (mainly from journal papers in MCD). These cases produced 135 entries for 48 Entrez Gene IDs (EGIDs). While, in general, only small numbers of compounds are linked to these non-canonical protein sequences (i.e. alternative splice forms of the UniProt or RefSeq sequences corresponding to the EGID), these are important to capture for pharmacological differences. The human EGID total in Additional file 1 is thus 1,654.

The summed number of compounds is 1,673,803. However, because the same compound may be assayed against different targets in the same or different documents, the unique set is 823,179. The average is 964 and the median 41 compounds-per-target. The top-278 proteins cover 90% of the total compounds at a cut-off of just over 1000 compounds-per-target. The summed number of documents is 53,440. The unique totals, 12,764 journal articles and 15,170 patents, are lower because of those that include results for more than one target. A subset of the top-50 targets with a cut-off just below 9,000 compounds-per-target is shown in Table 3. The binned distribution for the complete Additional file 1 is given in Table 4.

**Table 3:** Ranking of top-50 targets by numbers of compounds and documents.

Rank	Approved Symbol	Entrez Gene ID	No of compounds	No of documents
**1**	F10	2159	42869	690

**2**	CNR1	1268	29658	578

**3**	KDR	3791	27661	350

**4**	MAPK14	1432	24568	309

**5**	DRD3	1814	23405	508

**6**	F2	2147	22853	768

**7**	HRH3	11255	22748	407

**8**	TACR1	6869	21908	626

**9**	MMP13	4322	20590	315

**10**	CNR2	1269	19712	464

**11**	MMP1	4312	17525	394

**12**	ADORA2A	135	17181	532

**13**	EGFR	1956	16581	445

**14**	SLC6A4	6532	16571	403

**15**	MMP9	4318	16537	344

**16**	HTR6	3362	16457	504

**17**	MMP2	4313	16405	310

**18**	HTR2C	3358	15945	475

**19**	CRHR1	1394	15550	222

**20**	MC4R	4160	15084	299

**21**	HTR2A	3356	14622	509

**22**	NPY5R	4889	14547	216

**23**	CCR3	1232	14136	114

**24**	OPRM1	4988	13394	466

**25**	DPP4	1803	13057	308

**26**	REN	5972	12894	438

**27**	CALCRL	10203	12615	137

**28**	CTSS	1520	12426	177

**29**	CHRM3	1131	12398	412

**30**	CCR2	729230	12208	160

**31**	DRD2	1813	12050	564

**32**	MET	4233	11745	118

**33**	ADORA1	134	11644	480

**34**	GSK3B	2932	11283	198

**35**	CCR5	1234	11179	197

**36**	CXCR2	3579	10851	183

**37**	SRC	6714	10838	282

**38**	MCHR1	2847	10821	209

**39**	EDNRA	1909	10769	260

**40**	NR3C1	2908	10687	199

**41**	EDNRB	1910	10601	239

**42**	HTR1A	3350	10015	627

**43**	OPRK1	4986	9690	375

**44**	TACR2	6865	9676	301

**45**	SLC6A2	6530	9671	272

**46**	ADORA3	140	9533	457

**47**	OPRD1	4985	9500	394

**48**	HSD11B1	3290	9334	151

**49**	ELANE	1991	9173	308

**50**	TRPV1	7442	8988	150

**Table 4:** Binned distribution of compounds-per-target.

Compound bin	Targets above bin
10000	42

5000	95

2000	194

1051 (90% total)	278

1000	287

500	380

200	526

100	667

50	816

10	1194

2	1591

1	1736

This includes all entries in the Additional file 1.

Inspection of our results indicated, not unexpectedly, a correlation between the number of compounds and number of documents. However, this was a very broad distribution because the extraction averages (given in Table 1) of 14 compounds-per-paper and 44 compounds-per-patent, varied by at least one order of magnitude for the former and two orders of magnitude for the latter. In the following section target proteins will thus be referred to by their rank on the basis of compounds. Those within the top-50 are listed in Table 2 while any below these in the ranking are listed in Additional file 1. The triage we have used is stringent in that it maps 28% of the compounds and 22% of the documents in Table1. Consequently, it represents target-to-compound-to-assay mappings indicative of activity modulation of a single defined human protein target. Complex targets that cannot be resolved to a single EGID (e.g. the 20s proteosome) are not included.

### Content of *bona fide *Drug Targets

Detailed elaboration of what constitutes a drug target is outside the scope of this work but this has been reviewed [20]. We, as do most descriptions for sources of this type, use the term "target" broadly to encompass any compound-to-protein mapping in our large dataset. We consider the target figures and divisions given by TTD to be a good approximation (they include a proportion of authenticated one-to-many mappings) to a set of *bona fide *primary targets (i.e. where the interaction *in vitro *is mechanistically causative for the therapeutic effect *in vivo*). It should be noted that, without inspection of the individual documents or "prior knowledge", it is difficult to discriminate within database records *per se *between a *bona fide *drug target, a protein assay included for the purpose of discerning compound selectivity, off-target effects or modulating multiple targets with the same compound (i.e. polypharmacology) [21]. This classification problem is encountered for any large-scale collation of compound-to-protein mappings. It cannot be discerned clearly enough to be specified in the TCD database records because, while journal authors will typically explain the context and objectives of multiple assays, patent applicants often do not.

Nevertheless, it is clear from Table 2 that many of the top-50 proteins are not (yet) successful targets of approved drugs. A formal test was applied by determining the gene symbol intersect between Table 2 and the 185 targets of approved oral drugs from 2006 [2]. Despite there being some new targets for post-2006 approved drugs the result was only 23 in common, indicating that a high compound ranking *per se*, is not necessarily a predictor of successful approval. The targets-in-common across the entire list were 160. Inspection of the 25 targets not matched indicated that, in most cases, the primary literature either included assay data from non-human proteins (e.g. mouse or rat) or that the cell-based receptor pharmacology assays were not classed as "type B". One interesting exception is what could be classified as orphan target, tyrosine-3-hydroxylase, TH [Swiss-Prot P07101]. While a drug was approved for it, α-methyl tyrosine (CID 441350) many decades ago to treat pheochromocytoma, this is now rarely used because of side effects. Consequently, this protein identifier has not been linked to new research compounds within this set of extracted journal papers and patents.

### Cross-screening and Para-targets

The difficulty of discriminating primary targets from cross-screening activities is illustrated at the top of Table 2 for factor X, F10 [Swiss-Prot P00742] and thrombin, F2 [Swiss-Prot P00734]. They are not only the individual primary targets for the development of therapeutic protease inhibitors but also, because they are related as paralogues with a high sequence similarity and biochemical functions, they are typically chosen as cross-screening targets for each other. They can thus be termed "para-targets". We confirmed the extent of cross-screening by determining that there were 13,504 compounds-in-common and 357 documents-in-common (i.e. containing both thrombin and factor X inhibition data). This has the effect of pushing each of them higher in the compounds-per-target ranking. The second ranked para-target pair in Table 2 is the cannabinoid receptors 1, CNR1 [Swiss-Prot P21554] and 2, CNR2 [Swiss-Prot P34972] ranked at positions 2 and 10 respectively. These have 11,818 compounds-in-common and 342 documents-in-common. However, there is a difference for this pair in that antagonists have been predominantly pursued for CNR1 but agonists for CNR2 [22]. In addition, CNRI provides an example of screening data derived from a specific splice variant with unique pharmacological profile, designated as cannabinoid receptor 1B [Swiss-Prot P21554-3] ranked at 1216 [23]. Other para-target pairs illustrate different aspects. For the beta amyloid cleaving enzymes BACE1 [Swiss-Prot P56817], and BACE2 [Swiss-Prot Q9Y5Z0] clearly the former, ranked at 52, is the primary target but there is also some cross-screening for BACE2 ranked at 338. Another series of paralogues in the table, in order of compound ranking, are Cathepsin S, CTSS [Swiss-Prot P25774], Cathepsin K, CTSK [Swiss-Prot P43235], Cathepsin L, CTSL1 [Swiss-Prot P07711] and Cathepsin G, CTSG [Swiss-Prot P08311]. These are all cysteine proteases being explored for different diseases but are, as one might expect, extensively cross-screened for selectivity [24].

### Anti-targets

The first anti-target (i.e. cross-screening for potential liabilities in development) in the list, ranked at 83, is the hERG Kv11.1 potassium channel, KCNH2 [Swiss-Prot Q12809]. This is unsurprising considering the importance of checking for hERG inhibition [25]. Another anti-target is the drug efflux pump, ATP-binding cassette, sub-family B (MDR/TAP) member 1, ABCB1 [Swiss-Prot P08183] ranked at 313. However, a recent analysis suggests that, while an anti-target for anticancer agents, it can also be classified as a drug target for non-sedating antihistamines [26].

### Non-targets

The first non-target (i.e. without an established therapeutic context) is Trypsin, PRSS1 [Swiss-Prot Q3SY19], ranked at 114, because of its use as a mechanistic exemplar for cross-screening serine protease inhibitors. A second non-target, ranked at 261, is Albumin, ALB [Swiss-Prot P02768]. This is due to the routine testing of development compounds in albumin binding assays. Strictly speaking, this protein has no activity modulation but the compounds are nonetheless "mapped" in the binding sense. Slightly below this, at rank 295, is the amyloid beta A4 precursor protein, APP [Swiss-Prot P05067]. As an intact protein it is a non-target but inspection of the documents reveals two distinct strategies for compound testing. The first is the use of assays that measure down-regulation of APP production in cell lines. While this is clearly a therapeutic option to reduce amyloid peptide deposits, there is no data to suggest that the active compounds are actually binding APP. The second document set specifies beta amyloid aggregation antagonists (i.e. the peptide could be considered the target). Optimisation of these compounds would have the same therapeutic objective but would show different SAR. While some type of mechanistic splitting terminology for this target classification problem could be considered, it is important to note that the use of the APP identifier has at least facilitated data capture.

### Failed Targets

Target names can be recognised in the list where compounds in Phase III trials have been publically declared as either having safety concerns or did not show efficacy. An example of the former, the cannabinoid receptor 1, CNR1 [Swiss-Prot P21554] is ranked second but the clinical trial results for rimonabant (CID 104850) precluded approval because of an increased risk of depression and suicide [27]. During the initial drafting of this manuscript we selected the cholesterol ester transfer protein, CETP [Swiss-Prot P11597], ranked at 263 as a failed target example because the progression of torcetrapib (CID 159325) was halted [28]. However, within months, there was a more successful phase III outcome for anacetrapib (CID 11556427) targeting the same protein [29]. Thus, the extent to which late-stage failures constitute de-validation remains an open question, given not only that some of those targets can still "make it" but also that efficacy in a pharmacogenetically stratified cohort or repurposing for an alternative indication might still be achievable.

Nevertheless, the ability to flag likely de-validation in the listing we have produced would be valuable. However, the capture of historical data has the limitation that targets can achieve a high ranking if many compounds have been generated during validation and proof-of-concept studies even where these eventually fail. In addition, negative data produced during the research phase is less likely to be published. Our data can be analysed on a per-year basis, so the observation of a sustained decline in compounds (i.e. less publications on that target) can infer that validation has stalled (data not shown).

### Tractability Assessment by Molecular Frameworks Analysis

The upper part of our compounds-per-target distribution (Table 2 and Additional file 1) provides a *de facto *chemical tractability ranking. The term is used here as a measure of the probability that a useful level of potency for chemical modulation of the therapeutically relevant biochemical activity of a protein can be readily achieved *in vitro*. While this is likely to be related to the HTS primary hit-rate, it must be remembered that a high proportion of the compounds in MCD and TGD have gone through some hit-to-lead optimisation. We thus choose to differentiate, on a target basis, between chemical tractability and druggability. We consider the latter to be the likelihood of developing compounds with appropriate *in vivo *bioavailability, efficacy and safety profiles [30]. These two characteristics are usually related because high chemical tractability facilitates the generation of more compound series *in vitro *which, in turn, provide more optimisation options *in vivo*. The main caveat with ranking targets just by compound numbers (as in Table 3) is that, in order to be useful, a tractability metric needs to factor-in the chemical diversity of the compound set. For example, targets mapped to large numbers of highly similar analogues might actually be less tractable than those with smaller absolute compound numbers but covering a broader range of chemotypes.

We have consequently exploited the compound listing to produce a detailed assessment of chemical diversity by comparing molecular frameworks and scaffolds on a per-target basis. These are well-developed concepts in medicinal chemistry and there are a number of ways in which chemical structures can be abstracted. An approach, initially described by Bemis and Murcko [31], considers such frameworks as a collection of ring systems connected by linkers, after removing side chains. A more detailed hierarchy was used by Xu and Johnson [32] to define Molecular Equivalence Indices (MEQIs) as tools for molecular similarity measures. These approaches have been used for classifying and visualising compound collections [33,34], scaffold-hopping [35], comparing small sets of bioactive molecules [36] and large vendor libraries [37], target selectivity [38] and to differentiate between drugs, clinical candidate and bioactive molecules [39].

For our analysis we generated five levels of frameworks and scaffolds using software developed at GVKBIO:

1. Molecular Framework 1 (MF1): This is generated from the normalised molecular structure by removing all terminal side chains. Exocyclic double bonds (atoms connected to ring systems through multiple bonds) and double bonds directly attached to the linker are kept.

2. Molecular Framework 2 (MF2): This is derived from MF1 by removing exocyclic double bonds and double bonds directly attached to the linker.

3. Carbon Scaffold (CS): This is derived from MF2 by ignoring all atom types other than Carbon.

4. Atom Type Scaffold (ATS): Also derived from MF2 but ignoring bond types.

5. Graph Scaffold (GS): Also derived from MF2 but ignoring bond types or atom types.

**Figure 2:**
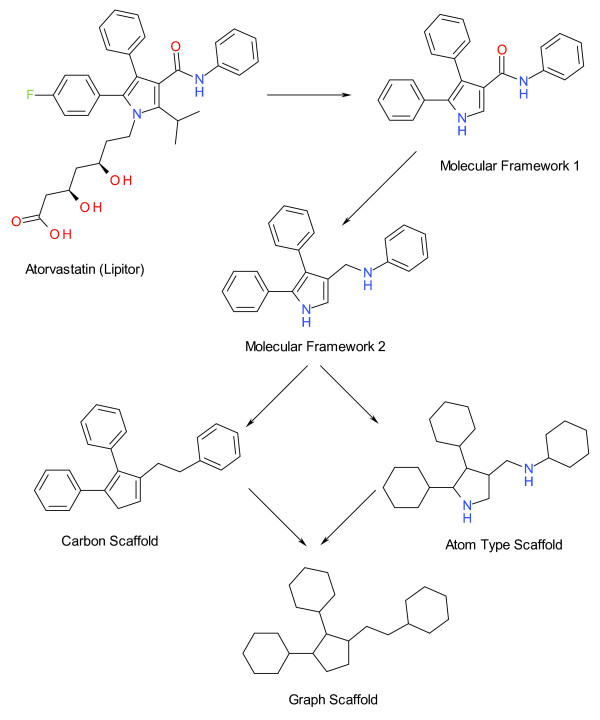
**The molecular topology hierarchy exemplified for Atorvastatin (Lipitor)**.

An example of the five levels of molecular topology hierarchy is shown for atorvastatin (CID 60823) in Figure 2. We applied these abstractions to the entire compound set, on a per-target basis, and the results are included in Additional file 1. The availability of the molecular topology breakdown allows target tractability to be examined in alternative ways. We have made a comparative top-20 ranking at three levels, total compounds, MF2 and GS, in Table 5.

**Table 5:** Top-20 target rankings by compound count and molecular frameworks.

Cmpd ranking	Target symbol	MF2 ranking	Target symbol	GS ranking	Target symbol
1	F10	1	F10	1	F10

2	CNR1	7	HRH3	7	HRH3

3	KDR	2	CNR1	3	KDR

4	MAPK14	6	F2	6	F2

5	DRD3	3	KDR	2	CNR1

6	F2	5	DRD3	24	OPRM1

7	HRH3	28	CTSS	20	MC4R

8	TACR1	8	TACR1	5	DRD3

9	MMP13	10	CNR2	8	TACR1

10	CNR2	12	ADORA2A	29	CHRM3

11	MMP1	4	MAPK14	28	CTSS

12	ADORA2A	29	CHRM3	26	REN

13	EGFR	9	MMP13	10	CNR2

14	SLC6A4	26	REN	4	MAPK14

15	MMP9	24	OPRM1	12	ADORA2A

16	HTR6	23	CCR3	31	DRD2

17	MMP2	20	MC4R	38	MCHR1

18	HTR2C	17	MMP2	37	SRC

19	CRHR1	11	MMP1	16	HTR6

20	MC4R	25	DPP4	49	ELANE

Columns 1 and 2 are the compound count ranking, columns 3 and 4 show the molecular framework 2 (MF2) ranking and colums 5 and 6 show the graph scaffold (GS) ranking.

We can see that the metalloprotease MMP1 drops from its original compound ranking at 11 down to 19 when ranked by MF2. The cathepsin CTSS moves in the opposite directed from 28 in the original ranking up to 7 by MF2. In the GS ranking we see the elastase ELNA rising from 49 to 20 but the kinase MAPK14 dropping from 4 to 14. Thus, for an individual target the tractability depends significantly on the molecular framework level used for ranking.

**Figure 3:**
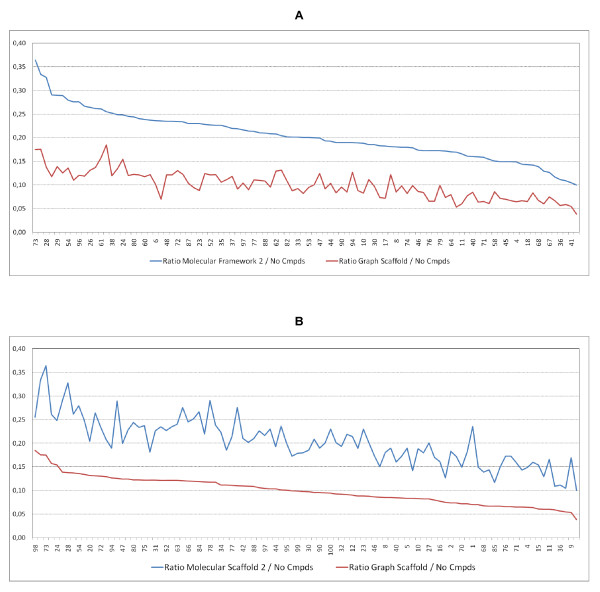
**Sorted MF2 to number of compounds ratio (a) and Graph Scaffold to number of compounds ratio (b)**. This is plotted for all targets with more than 4869 compounds from Additional file 1.

The MF2 level is particularly relevant for medicinal chemistry because it represents a practical scaffold level from which substituents can be permutated for the preparation of analogue series or compound libraries for SAR studies. For this reason, we have extended the analysis in Table 5 by plotting top-100 targets from Additional file 1 (corresponding to 4680 upwards compounds-per-target) in Figure 3.

More compounds with fewer MF2 scaffolds indicate lower tractability (e.g. an MF2: compound ratio of 0.13 for ESR2 from a total of 6,695 compounds). A larger ratio indicates higher tractability (e.g. 0.36 for HDAC1 from a total of 6,124 compounds). We suggest this complements the ranking by compounds alone and, in this case, clearly differentiates the relative rankings of 67 for ESR2 and 73 for HDAC1.

The molecular scaffold results can be conceived as collapsing the ensemble of structures mapped to a target in progressive stages of abstraction. Thus, moving from MF2 and GS we see a reduction as more compounds collapse into the latter. The target trends in Figure 3 are different for MF2 and GS. In addition, the spiked shape of the abstractions show these can be highly target-specific. As an example of utility, the visualisation of the chemotype landscape for targets with very large compound sets (e.g. over 10,000) is much easier when the GS ring-type abstractions can be displayed and browsed.

The utility of using public data for examining tractability before embarking on drug discovery project directed against targets and the correlation with ligand-based experimental assessments has recently been pointed out [40].

## Conclusions

We have triaged a commercial database to provide human target protein identifiers ranked by the numbers of compounds linked to them via direct biochemical assay data and the numbers of documents from which these associations were extracted. As far as we are aware, this is the largest published listing of this type and presents a detailed assessment of the major part of the human molecular target landscape that has been, or is, under active investigation [41]. The unique of scale of this is exemplified by comparing the equivalent compound-to-target count for F10 in ChEMBL of 5,871 against 42,869 in this work. This is because the process includes the extraction compounds and data not only from journal articles but also from patents.

Nevertheless, there are limitations (beyond our triage choices) that preclude this being a complete capture of the available data. The first is that in the PubChemBioAssay database, while the direct assay methods may have been published as documents, the compound structures, protein identifiers and result sets are only instantiated in *silico *[42,43]. The second limitation is the necessity to cap the number of examples extracted from a patent. The third is that patent data extraction is currently limited to the "big ten" target classes and English language applications (but efforts are underway at GVKBIO to expand this). The fourth is journal selection as opposed to all journals. Whilst these pragmatic constraints may bias the extractions, we propose that, in SAR terms, they are selective for higher quality data.

Our complete set of results include many proteins that would not be considered *bona fide *drug target candidates, not only for the reasons already pointed out in the review of the list, but also by being in the tail of the compound distribution. However, the inclusion of even the singletons (one compound from one publication) is useful not only because they have been authenticated by expert extraction but also both the target and the compound may have a wider set of relationships using different species and/or assay type restrictions. Imposing any cut-off for "target likelihood" is clearly arbitrary but taking, a lower limit of 20 compounds-per-target still covers just over 1000 proteins. This brings it into congruence with the data-supported target count of 836 human proteins for which moderately potent small-molecule chemical starting points had previously been reported [44].

Our breakdown of the compound sets into molecular scaffolds provides a useful measure of target-specific chemical tractability. Nevertheless, we can point out factors that may be skewing the ranking upwards. The first is the cross-screening effect already mentioned where many compounds mapped to a target are not being optimised for that target. A second effect is that resources assigned to target projects are determined by factors such as market potential, competitive positioning and unmet clinical need. This skews the distribution away from an objectively neutral ranking of tractability *per se *towards those targets the research community is collectively "working hardest" on. This intense focus also produces patent thickets (in the sense that many of the synthetically feasible chemotypes and analogues that can bind to a particular active site have already been claimed) that will also drive the expansion of chemical diversity for popular targets.

Readers are encouraged to explore their own additional analyses for Additional file 1. These could include generating intersects and differences with, for example, disease associated protein lists or other target protein lists extracted from public databases. In addition, the proteins could be further divided by sub-family and/or the existence of representative 3D structures in PDB. Further large-scale studies of the target landscape analogous to those reported here will be important as drug discovery continues to expand towards new therapeutic areas, new targets, broader cross-screening activities, repurposing and polypharmacology.

## Endnotes

Protein designations first used in the text are given as their common name followed by the HGNC approved human gene symbol as used in the result tables. These are followed by the Swiss-Prot ID. Drug names are accompanied by their PubChem compound identifiers (CIDs).

## Competing interests

AstraZeneca and GVKBIO have a business relationship.

## Authors' contributions

The study was conceived by CS, SM and SARPJ. The data was generated by KB and the manuscript drafted by CS and SM. All authors read and approved the final manuscript.

## Supplementary Material

Additional file 1 **Additional material**. A list of proteins with names, symbols and Entrez Gene identifiers (Microsoft EXCEL). It also includes compound and document counts and the molecular framework breakdown for the compound sets. Click here for file
